# 

**Published:** 1885-10

**Authors:** 


					﻿OLD -SERIES
Xw. xkv

fclCAl^BW
® JdurnaESb.

ty" W.F.WESTMORELAND, wg
H.V.M.MILLER. M.D.LL .D.>
JAMES.A.GRAY. M.D.'
MjBUSHED BY JAMES.P.HARRIS0N&.CQ3
wpAtlanta ,ga. £a
SUBSCRIPTION: $2.50 A YEAR.
				

